# Coronal Streamer Brightness Profiles Investigated with BriLo Using Parker Solar Probe White-Light Data

**DOI:** 10.1007/s11207-025-02601-1

**Published:** 2026-01-14

**Authors:** Greta M. Cappello, Manuela Temmer, Andrea Lienhart, Giuseppe Nisticò, Guillermo Stenborg, Mark G. Linton, Yara De Leo, Stephan G. Heinemann, Paulett C. Liewer, Russell A. Howard, Volker Bothmer

**Affiliations:** 1 Institute of Physics, University of Graz, Graz, Austria; 2 Department of Physics, University of Calabria, Cosenza, Italy; 3 Applied Physics Laboratory, Johns Hopkins University, Baltimore, USA; 4 Naval Research Laboratory, Washington, DC USA; 5 National Institute for Astrophysics, Astrophysical Observatory of Catania, Catania, Italy; 6 Jet Propulsion Laboratory, Pasadena, CA USA; 7 Institut für Astrophysik und Geophysik, Georg-August-Universität, Göttingen, Germany

**Keywords:** Coronal streamers, Thomson scattering, White-light observations, K-corona, Parker solar probe

## Abstract

We present the Brightness–Location (BriLo) method, a novel single-spacecraft technique which exploits the Thomson scattering theory for localizing extended coronal features such as streamers using white-light (WL) imaging. Beyond determining the longitude and latitude of coronal features, the method also provides estimates of their geometrical properties, such as angular width (column depth). Validation is performed through geometrical triangulation with multi-viewpoint coronagraphs (the Solar TErrestrial RElations Observatory A COR2 and the Solar and Heliospheric Observatory C2–C3). The method is applied to ten coronal streamers observed by the Wide-Field Imager for Solar Probe (WISPR) on board the Parker Solar Probe (PSP) between encounter 1 – 17. We applied BriLo to two different data products, L3 and LX, which differ in K-corona treatment and absolute brightness levels. The L3 and LX results show good agreement in deriving streamer directionality, with differences of 2 – 30° in longitude and 1 – 6° in latitude. Both datasets provide longitude and latitude estimates that are broadly consistent with triangulation results. We further classified streamers and compared their locations with potential-field source surface (PFSS) extrapolations of the heliospheric current sheet (HCS). Helmet streamers are generally found close to the HCS, whereas pseudostreamers in proximity to active regions. In conclusion, the application of BriLo to LX data yields realistic streamer widths of several to ten degrees, while L3 data produce unrealistically narrow values below one degree. This discrepancy arises from the line of sight (LOS) integration of the observed signal and the dependence of F-corona removal on background estimation and coronal conditions. Overall, BriLo proves to be a robust tool not only for streamer localization but also for assessing and validating WL imaging techniques.

## Introduction

It is well established that changes in the photospheric magnetic field, such as the emergence of new magnetic flux or its redistribution by surface flows, shape the coronal features. Magnetic field variations are also reflected in white-light (WL) observations, revealing changes in the morphology and dynamics of coronal structures (Wang et al. 1997). In that regard, coronal streamers are large-scale structures believed to trace the global structure of the solar magnetic field (e.g. Howard and Koomen 1974; Hansen, Sawyer, and Hansen 1974) forming what is commonly referred to as the “streamer belt” (e.g. Crooker et al. 1996) and mapping the outflow of slower, denser solar wind. The streamer belt is therefore the plasma manifestation of the Sun’s global magnetic field structure.

Helmet streamers form above magnetic fields of opposite polarity, creating a closed magnetic field region below a cusp-shaped structure (e.g. Rušin et al. 2010). They are characterized by a bright dome composed of closed magnetic field lines that separate the opposite magnetic polarities, forming an extended “helmet” shape, a heliospheric plasma sheet (HPS) and the heliospheric current sheet (HCS). On the other hand, pseudostreamers are characterized by a closed magnetic field that separates same radial polarity magnetic regions in the photosphere. These same polarity radial magnetic fields come together, also in a cusp shape, above the closed field region of the pseudostreamer (Wang et al. 2007; Riley and Luhmann 2012). But as they have the same sign of radial magnetic field, they do not create a current sheet in the heliosphere where they come together. In contrast to pseudostreamers, helmet streamers cover a magnetic polarity reversal which sometimes is related to a magnetic sector boundary crossing at 1 AU (Crooker et al. 2012). Both helmet streamers and pseudostreamers show extended rays in coronagraph images, however, the bright dome above $\sim 2$ solar radii is missing for pseudostreamers (see Wang et al. 2007).

Although the slow solar wind is commonly observed near the HCS and is often associated with the helmet streamer belt in WL observations, its solar sources are not necessarily confined to low latitudes (Abbo et al. 2016, and references therein). Particularly during solar minimum, magnetic field lines originating near the boundaries of polar coronal holes can funnel way down to the helmet streamer belt near the solar equator (e.g. Gosling et al. 1997; Zhao, Zurbuchen, and Fisk 2009; Xu and Borovsky 2015). The S-web model refines this picture. According to this model, the intrinsic complexity of the photospheric magnetic field gives rise to a network of narrowly connected and disconnected coronal holes of the same polarity that remain magnetically linked. This configuration generates a separatrix web in the heliosphere, effectively broadening the source region of the slow wind to include areas significantly offset from the HCS (Antiochos et al. 2011).

During low solar activity periods the coronal streamer belt is confined to low latitudes (typically below 20 – 30°) near the solar equator (Vsekhsvyatsky et al. 1965) and the HCS appears to be flat, reflecting the rather simple magnetic field conditions on the Sun (Riley, Linker, and Mikić 2002). However, as the solar activity rises, the streamer belt is found over a broader latitude range and coronal holes appear in the equatorial regions (Harvey and Recely 2002), making the HCS strongly tilted and warped (Wang et al. 2000; Riley et al. 2006).

Coronal structures in WL data are observed through sunlight that is Thomson scattered by free electrons, known as the K-corona (see, e.g., Koutchmy et al. 2004). Therefore, their brightness depends on both the electron density and the scattering geometry (Vourlidas and Howard 2006). Hence, the morphology of streamers is dominated by the Thomson scattering process and gives rise to changes in their appearance due to the distribution of the structures on the Sun (e.g. Wang et al. 1997, 2009). Ambiguity about streamer morphology, such as its width and density remains due to line of sight (LOS) effects and viewing geometry.

Liewer et al. (2001) determined the 3D positions of eight coronal streamers and verified that most of the streamers were located near the HCS or active regions. Thernisien and Howard (2006) and Saez et al. (2005) showed that the fine structures observed in the coronagraph images, such as double or triple stalks, arise from folds in the HCS. In particular, a fold is formed by undulations of the HCS, and stalks and streamers appear in the WL images in locations where the undulating HCS is locally parallel to the LOS of the observing instrument, thus creating more of a scattering signal because more of the HCS is along that LOS, and the HCS (or at least the HPS) is usually denser than the surrounding heliosphere. Since 2018, the Wide-field Imager for Parker Solar Probe (WISPR; Vourlidas et al. 2016) on board Parker Solar Probe (PSP; Fox et al. 2016) has revealed an increasingly rich and dynamic variety of coronal structures, offering unique insights into the evolving solar corona (Raouafi et al. 2023). Poirier et al. (2020) used WISPR to identify multiple substructures within streamers and pseudostreamers, demonstrating its ability to resolve the fine-scale structure of the densest parts of streamer rays. Liewer et al. (2023) investigated several rays detected with WISPR during perihelia at encounter eight (E8; closest approach 16 $\mathrm{R}_{\odot}$) and E11 (13 $\mathrm{R}_{\odot}$), revealing large variations in size and brightness found with longitudes along the streamer belt plasma. The latter seems therefore to be highly variable, reflecting back on the slow solar wind outflow (see also Morgan and Cook 2020). Hence, when investigating coronal structures, the linking to their source regions for interpreting generation mechanisms or brightness variations is of high importance.

In our study, we present the Brightness–Location (BriLo) method for determining the longitude and latitude of coronal streamer structures on the Sun from single-spacecraft observations, as well as for deriving their physical properties (e.g., width) using the Thomson scattering theory. The method is based on the theoretical framework provided by Nisticò et al. (2020). They demonstrated that the temporal profiles of total brightness of dynamic plasma structures, such as outward propagating simple coronal mass ejections or blobs, depend both on the heliocentric distance of the feature and Thomson scattering angle. We adapted the time-dependent method described by Nisticò et al. (2020) such as to analyze total brightness profiles of extended, quasi-static coronal streamers at a fixed time. Using WISPR observations we provide an easy-to-use method to derive the 3D location of coronal streamer structures by simply measuring their brightness profiles as function of the central angle, defined as the angle between the streamer ray and the Sun to PSP ray. We then relate the derived streamer coordinates to the HCS position as extracted from potential field source surface (PFSS) extrapolations at 2.5 $\mathrm{R}_{\odot}$. Besides estimating the location of extended streamer features, the theoretical brightness profile includes parameters that characterize the streamer density and LOS width, allowing us to infer geometric and structural information from single spacecraft observational data. In addition, the study shows limitations of current image processing techniques used to isolate the K-corona signal in WL data.

The article is organized as follows: Section 2 describes the data and methodology, Section 3 presents our results, Section 4 discusses their implications and summarizes our findings with prospects for future work.

## Data and Methods

In our study we focus on data from the WISPR telescopes on board the PSP spacecraft. The WISPR suite consists of two telescopes, the “inner” (hereafter WISPR-I) and the “outer” (hereafter WISPR-O). Their combined field of view (FoV) ranges from about 13.5° to 108° elongation, and covers 40° in the transverse direction. Both telescopes observe the solar corona in WL, their passbands being about 470 – 800 nm (Vourlidas et al. 2016; Hess et al. 2021; Wood et al. 2022). The two main components of the recorded signal are from (1) the K-corona, which results from the Thomson scattering of photospheric light by the free electrons in the corona; and (2) the F-corona, which arises from the photospheric light scattered by the dust particles in orbit around the Sun. The calibrated WISPR images (hereafter, the L2 data set) are expressed in mean solar brightness (MSB) units (Hess et al. 2021).

The physics of Thomson scattering has been extensively developed over the last century (e.g. Jackson and Fox 1999), with foundational treatments provided by Minnaert (1930), van de Hulst (1950), and Billings (1966). A key concept introduced by Vourlidas and Howard (2006) is the Thomson surface (TS): a spherical surface centered halfway between the Sun and the observer, with a diameter equal to their distance. The observed brightness strongly depends on the scattering angle, $\chi $, which is defined as the angle between the observer’s LOS and the radial vector from the Sun to the scattering electron. For any given LOS, the scattered emission is maximized where the scattering angle is perpendicular to the LOS, i.e., at $\chi = 90^{\circ}$, which corresponds to the location of the TS. The observed enhancement arises because the incident photon flux and the average electron density along the LOS (i.e., assuming that the average density profile is spherically symmetric and decreases with distance from the Sun) are highest at this location. The scattering efficiency, which quantifies how efficiently an electron scatters a photon into the $\chi $ direction, is given by $1 + \cos ^{2} \chi $. Along a specific LOS, $\chi $ varies from $0^{\circ}$ to $180^{\circ}$, so the scattering efficiency ranges from a maximum of 2 at $\chi = 0^{\circ}$ and $180^{\circ}$ to a minimum of 1 at $\chi = 90^{\circ}$. Consequently, a higher incoming photon flux and a greater density of scattering electrons both contribute to maximizing the scattered emission at $\chi = 90^{\circ}$.

Since both the K-corona and the F-corona signals are present in the L2 images, the latter must be removed to isolate the K-corona signal. Due to the nature of the signal in the L2 images and the orbital characteristics of the spacecraft, the background difference imaging techniques, developed for WL imagery at 1 AU, cannot be used. Therefore, the WISPR team implemented several approaches to minimize the effect of the F-corona background. Among them, the so-called L3 technique, as outlined in the Appendix of Liewer et al. (2023), is particularly effective to reveal the large-scale K-corona structures at the expense of blurring the surrounding medium.

To avoid this caveat, other WISPR-customized techniques were developed (hereafter, the LX and LW techniques; Stenborg, Vourlidas, and Howard 2025). Unlike L3, the LX approach exploits the time domain over long time windows ($\geq 1$ year) to model the F-corona background while minimizing the influence of the variable K-corona. This preserves long-lived and quasi-stationary features, making it particularly well suited for the objectives of the present study. In contrast, the LW method, along with other short-term time-domain processing techniques (e.g. Alzate et al. 2021; Alzate and Di Matteo 2025), enhances transient features by estimating and subtracting a background over short running windows (typically a few hours to 1 day), thereby suppressing stationary coronal structures. A detailed comparison of LX and LW, including their respective strengths and limitations for different scientific applications, is presented in Stenborg, Vourlidas, and Howard (2025).

### Streamer Tracking

The aim of this study is to introduce a novel methodology to derive the 3D orientation (i.e., longitude and latitude) of coronal streamers. The angular coordinates are expressed in the heliographic Stonyhurst (HGS) reference system. For the present analysis, we selected by visual inspection 10 coronal streamers observed in WISPR-I images obtained during PSP solar encounters^1^ E1 through E17 (more details in Section 3).

**Figure 1:**
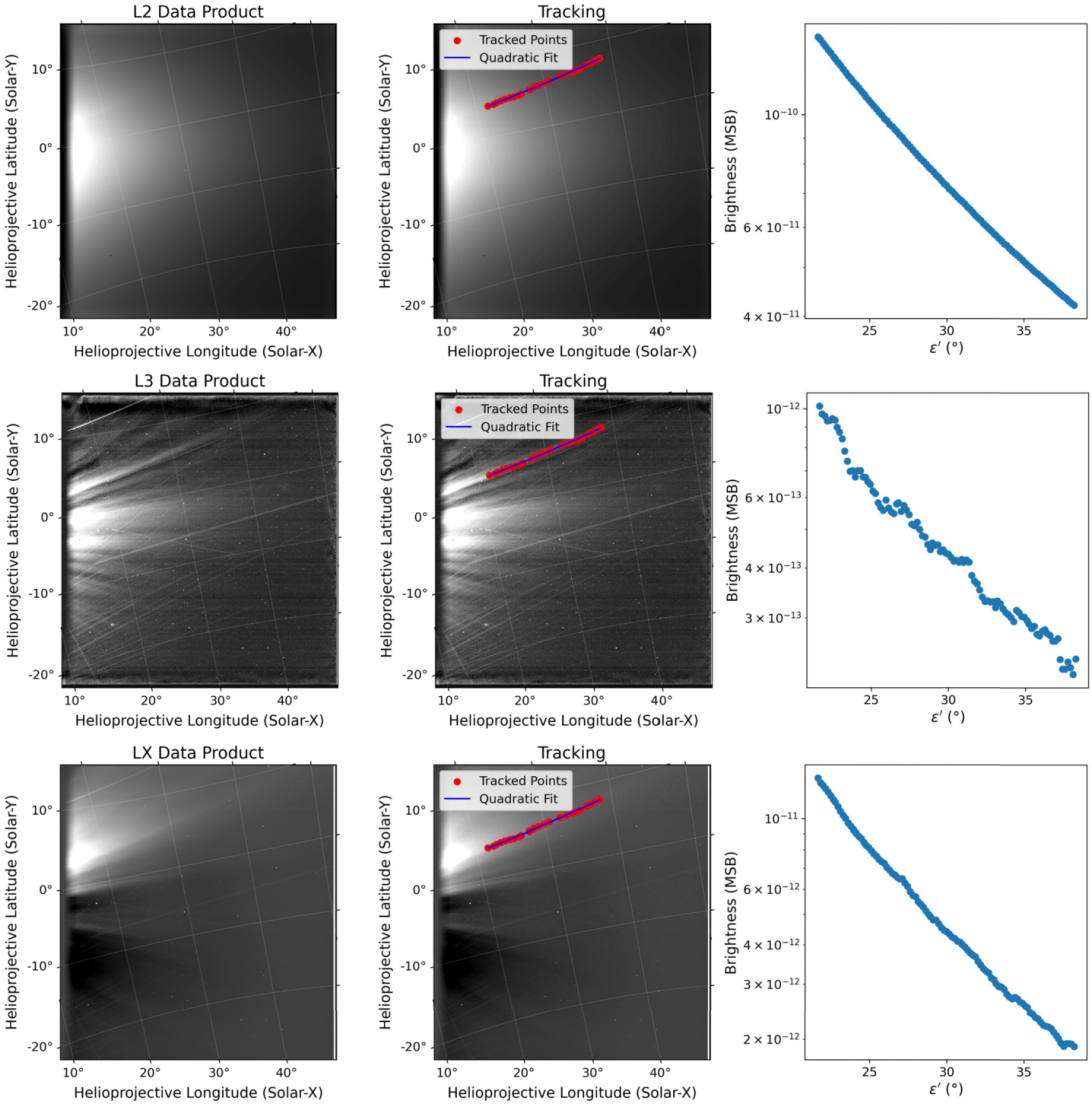
Example of a coronal ray tracked in WISPR-I data from November 19, 2021, at 15:03:25 UT on the basis of L2 (top row), L3 (middle row), and LX (bottom row) images. Left panels show the plain WISPR data product. Middle panels show the tracking of a streamer with the selected points (red dots) on which a quadratic fit (blue line) is applied. Right panels present the corresponding brightness profiles extracted from the track as a function of central angle (see Section 2.2).

For illustration purposes of our approach, in the top left panel of Figure 1 we display an L2 WISPR image obtained on November 19, 2021 (E10), when PSP was at 25.3 $\mathrm{R}_{\odot}$. The middle and bottom panels of the left column show the corresponding L3- and LX-processed versions, respectively. The grid is in a helioprojective cartesian (HPC; Thompson 2006) coordinate system, which is an observer-centered system wherein the Sun is at $[0,0]$ deg (longitude and latitude).

The L2 data products are strongly affected by the F-corona signal which dominates at heliocentric distances beyond $\sim 3\,\mathrm{--}\,4~\mathrm{R}_{\odot}$ (Koutchmy and Lamy 1985). As such, the L2 image shows a diffuse brightness distribution with no clear signature of any discrete structure. On the other hand, the L3-processed image clearly shows several coronal rays by having minimized the contaminant effect of both the F-corona and the surrounding diffuse, K-corona components. On the other hand, as can be seen in LX-processed version (bottom panel of the left column), the coronal rays are seen embedded in the surrounding K-corona environment.

The rays analyzed in this study are tracked in L3 data (i.e., where streamers are observed best) at distances ranging from 5 to 35.3 $\mathrm{R}_{\odot}$, depending on the individual case (rays 1 – 10), as summarized in Table 1. The middle column of Figure 1 displays a red line indicating a track along the spine of one particular streamer. The location of the track was determined in the L3 image by manually clicking at selected positions along the streamer. The data points selected along the streamer were then fitted with a second degree polynomial function to obtain a “smooth” track.

**Table 1:** Summary of BriLo and triangulation results for ten coronal streamers. Columns 1 – 3 list the streamer number, PSP encounter, and UTC observation time. Columns 4 – 6 give the PSP position in HGS coordinates. Columns 7 – 11 present BriLo results, with L3 as standard and LX included when available; $r_{2}$ is the heliocentric distance range of tracking points, and $\gamma '$ is the angle at the Sun in the PSP-Sun-P triangle (see Figure 2). Longitude and latitude of the ray are in the HGS frame. Columns 12 – 15 show triangulation results and the instruments used.

No.	EN	Observing date	PSP			Brightness-location method						Triangulation			
			*d* [$\mathrm{R}_{\odot}$]	long [°]	lat [°]	level	$r_{2}$ [$\mathrm{R}_{\odot}$]	*C* [MSB m^2^]	$\gamma '$ [°]	long [°]	lat [°]	long [°]	lat [°]	Ins. 1	Ins. 2
1	1	2018-11-06T15:40:53	35.87	112.4	−4.1	L3	[20.6,61.2]	$7.2^{+1.2}_{-1.6}\cdot 10^{7}$	$114^{+3}_{-5}$	$227.5^{+3.7}_{-5.4}$	$5.4^{+0.2}_{-0.3}$	229 ± 12	4.9 ± 0.4	STA/COR2	WISPR-I
2	7	2021-01-17T07:30:28	20.84	11.3	−3.1	L3	[14.5,61.3]	$8.1^{+2.9}_{-2.7}\cdot 10^{7}$	$124^{+4}_{-3}$	$136^{+4.3}_{-3.6}$	$-1.3^{+0.3}_{-0.6}$	127 ± 16	1.2 ± 1.1	LASCO/C3	WISPR-I
						LX	[9.1,21.4]	$1.4^{+0.1}_{-0.1}\cdot 10^{9}$	$97^{+3}_{-2}$	$108.8^{+3.6}_{-2.3}$	$-3.3^{+0.3}_{-0.5}$				
3	8	2021-04-29T14:27:17	16.23	−61.3	−3.8	L3	[7.0, 9.7]	$1.8^{+0.3}_{-0.4}\cdot 10^{7}$	$104^{+10}_{-17}$	$44.6^{+10.4}_{-17.7}$	$-17.5^{+1.2}_{-2.2}$	27 ± 6	−19.2 ± 0.4	STA/COR2	WISPR-I
						LX	[6.7,13.2]	$9.0^{+0.07}_{-0.03}\cdot 10^{8}$	$83^{+1}_{-2}$	$22.6^{+1.0}_{-2.1}$	$-19.9^{+0.2}_{-0.1}$				
4	9	2021-08-10T15:07:09	18.88	−126.3	−3.5	L3	[13.4, 102.1]	$1.7^{+0.54}_{-0.78}\cdot 10^{8}$	$125^{+5}_{-7}$	$359^{+5.3}_{-8.6}$	$-2.1^{+0.4}_{-0.8}$	350 ± 2	−4.9 ± 0.2	STA/COR2	WISPR-I
						LX	[8.9,28.7]	$1.7^{+0.23}_{-0.27}\cdot 10^{9}$	$107^{+4}_{-5}$	$342^{+4.3}_{-5.7}$	$-3.8^{+0.3}_{-0.3}$				
5	10	2021-11-19T15:03:25	25.29	−3.6	1.4	L3	[11.6, 64.8]	$5.9^{+3.5}_{-2.1}\cdot 10^{7}$	$110^{+17}_{-9}$	$107^{+17.4}_{-9.7}$	$6.6^{+0.9}_{-1.6}$	94 ± 12	11.2 ± 0.9	LASCO/C3	WISPR-I
						LX	[16.1,75.5]	$1.1^{+0.3}_{-0.15}\cdot 10^{9}$	$125^{+5}_{-3}$	$121.7^{+4.9}_{-2.9}$	$5.5^{+0.6}_{-0.7}$				
6	11	2022-02-25T09:45:19	13.68	−31.6	−2.9	L3	[11.0,55.7]	$7.8^{+0.6}_{-0.7}\cdot 10^{7}$	$129^{+1}_{-1}$	$101.3^{+1.0}_{-1.0}$	$-18.4^{+0.2}_{-0.1}$	89 ± 5	−20.5 ± 1.2	STA/COR2	WISPR-I
						LX	[8.4,27.4]	$2.2^{+0.1}_{-0.1}\cdot 10^{9}$	$119^{+1}_{-1}$	$91.2^{+1.0}_{-1.0}$	$-21.5^{+0.1}_{-0.1}$				
7	15	2023-03-18T05:30:15	14.22	−0.2	−3.8	L3	[7.2,17.2]	$1.9^{+0.33}_{-0.17}\cdot 10^{7}$	$99^{+8}_{-4}$	$101.3^{+9.1}_{-4.6}$	$-21.0^{+1.4}_{-0.7}$	${\scriptsize \begin{array}{l}72\pm 6 \\ 78\pm 5 \\ 72\pm 3\end{array}}$	${\scriptsize\begin{array}{l}-21.2\pm 0.8 \\ -21.5\pm 0.4 \\ -21.9\pm 0.7\end{array}}$	${\scriptsize\begin{array}{l}\mbox{LASCO/C2} \\ \mbox{LASCO/C2} \\ \mbox{STA/COR2}\end{array}}$	${\scriptsize\begin{array}{l} \mbox{STA/COR2} \\ \mbox{WISPR-I} \\ \mbox{WISPR-I}\end{array}}$
						LX	[11.0,36.0]	$1.2^{+0.2}_{-0.1}\cdot 10^{9}$	$121^{+2}_{-1}$	$124^{+2.6}_{-1.3}$	$-16.7^{+0.8}_{-0.3}$				
8	16	2023-06-23T07:30:15	20.07	−50.7	−2.2	L3	[7.4,12.7]	$1.7^{+0.03}_{-0.07}\cdot 10^{7}$	$85^{+1}_{-1}$	$32.6^{+1.9}_{-1.5}$	$29.9^{+0.1}_{-0.1}$	${\scriptsize\begin{array}{l} 28\pm 1 \\ 29\pm 1\end{array}}$	${\scriptsize\begin{array}{l}24.3\pm 0.7 \\ 26.0\pm 0.6\end{array}}$	${\scriptsize\begin{array}{l}\mbox{LASCO/C2}\\ \mbox{STA/COR2}\end{array}}$	${\scriptsize\begin{array}{l} \mbox{WISPR-I}\\ \mbox{WISPR-I}\end{array}}$
						LX	[15.1,56.0]	$5.0^{+1.2}_{-0.67}\cdot 10^{8}$	$132^{+3}_{-1}$	$85.5^{+3.3}_{-1.7}$	$23.3^{+0.7}_{-1.3}$				
9	16	2023-06-23T07:30:15	20.07	−50.7	−2.2	L3	[7.8,15.2]	$1.9^{+0.03}_{-0.07}\cdot 10^{7}$	$92^{+5}_{-6}$	$40.5^{+5.4}_{-6.5}$	$24.1^{+0.7}_{-0.4}$	${\scriptsize\begin{array}{l}29\pm 1 \\ 34\pm 1\end{array}}$	${\scriptsize\begin{array}{l}18.1\pm 0.9 \\ 19.7\pm 0.7\end{array}}$	${\scriptsize\begin{array}{l}\mbox{LASCO/C2} \\ \mbox{STA/COR2}\end{array}}$	${\scriptsize\begin{array}{l}\mbox{WISPR-I} \\ \mbox{WISPR-I}\end{array}}$
						LX	[11.4,37.7]	$2.1^{+1.0}_{-0.8}\cdot 10^{8}$	$117^{+9}_{-9}$	$75^{+4.3}_{-7.4}$	$20.9^{+1.2}_{-0.9}$				
10	17	2023-09-28T10:00:24	13.13	−172.9	−3.4	L3	[5.2,10.3]	$3.5^{+0.43}_{-0.37}\cdot 10^{7}$	$116^{+3}_{-3}$	$303^{+3.0}_{-3.0}$	$12.1^{+0.2}_{-0.3}$	${\scriptsize\begin{array}{l}308\pm 3 \\ 295\pm 10 \\ 318\pm 5\end{array}}$	${\scriptsize\begin{array}{l}10.9\pm 1.1 \\ 10.6\pm 0.3 \\ 10.3\pm 0.7\end{array}}$	${\scriptsize\begin{array}{l}\mbox{LASCO/C2} \\ \mbox{LASCO/C2} \\ \mbox{STA/COR2}\end{array}}$	${\scriptsize\begin{array}{l}\mbox{STA/COR2} \\ \mbox{WISPR-I} \\ \mbox{WISPR-I}\end{array}}$
						LX	[9.8,46.0]	$1.8^{+0.3}_{-0.2} \times 10^{9}$	$123^{+2}_{-1}$	$311^{+2.4}_{-1.7}$	$11.7^{+0.2}_{-0.3}$				

In the right column of Figure 1, we display the corresponding brightness profiles derived from each data product, L2, L3, and LX, along the track as a function of the central angle $\varepsilon '$ (see Section 2.2). Note that to minimize the detrimental effect of point-like sources as stars or bright linear features such as the signature of dust-spacecraft impact ejecta debris (Malaspina et al. 2022), we compute the median of the brightness over a $7\times 7$ wide box around each pixel along the track. The brightness profile from the L2 image is quite smooth, reflecting the brightness profile of the dominant F-corona. In contrast, the brightness profile in the L3-processed image reveals the subtle variations that arise from density inhomogeneities along the streamer, which are fully blurred in the L2 data. Note that the L3 brightness profile is about 2 orders of magnitude lower than the L2 profile (the L3 background subtraction not only removes the F-corona component but also a portion of the K-corona). The LX brightness profile instead shows the typical radial fall-off of a coronal streamer without the variability introduced by the small-scale inhomogeneities embedded in it.

### The BriLo Method

Nisticò et al. (2020) compared theoretical total brightness profiles with those obtained from ray-tracing simulations as a function of the elongation angle, $\varepsilon $, for features propagating within the orbital plane of PSP (more details in Figure 1 therein). They derived a time-dependent analytical expression for the total brightness $B$ [$\mathrm{Wm}^{-2}\,\mathrm{sr}^{-1}$] of a radially propagating feature (more details in Equation 2 therein) as a function of its longitude, propagation speed, and a parameter $C$, which encapsulates the physical properties of the structure, including the electron density $n_{e}$ and the width $h$ of the feature intersecting the LOS. This method has so far been applied only to simulated data; BriLo reproduces the approach by Nisticò et al. (2020), but adapts it for real WISPR observations of static structures in its field of view (FoV), which can also be located outside the PSP orbital plane.

**Figure 2:**
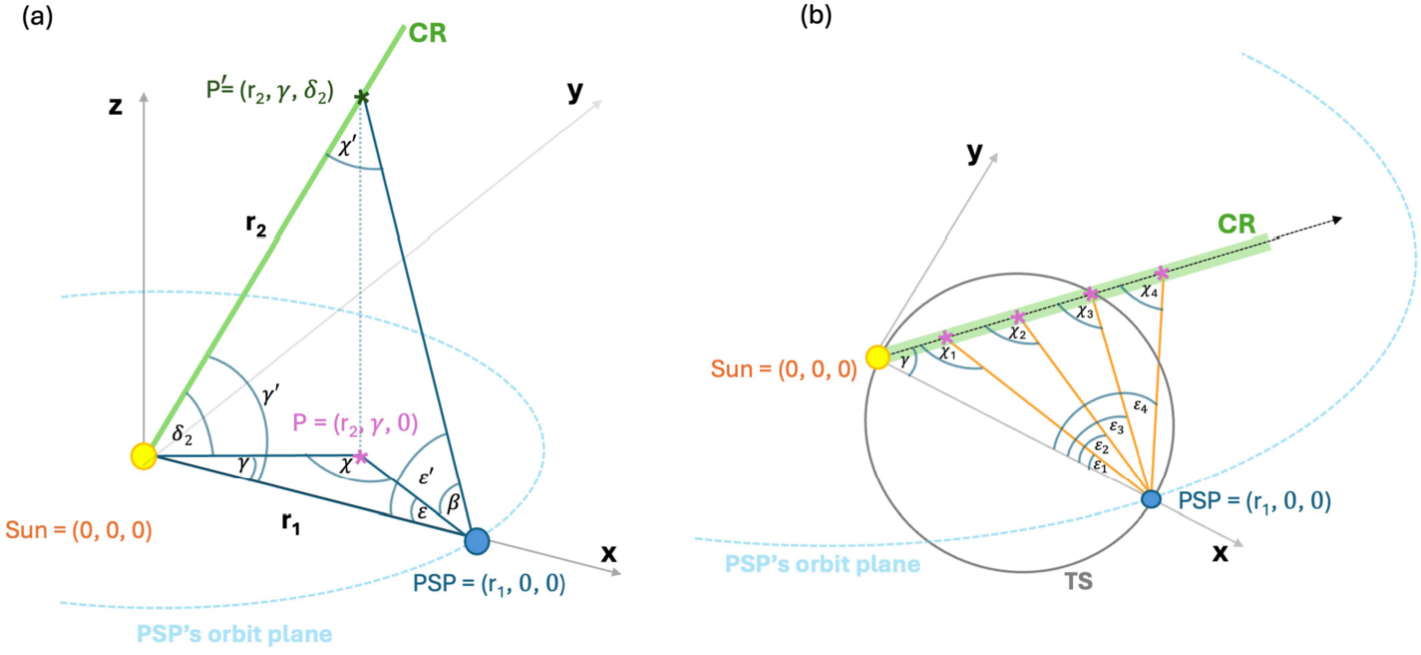
Representation of the 3D (panel a) and 2D (panel b) Thomson scattering geometry used in this work. In panel a, the coronal streamer is directed radially away from the Sun under the longitudinal angle $\gamma $ and latitudinal angle $\delta _{2}$. WISPR observes the coronal streamer over distance with a varying elongation $\varepsilon $, angle out of the orbit plane $\beta $, central angle $\varepsilon '$. A tracked point, P′, along the coronal ray is indicated with a dark green cross. P is the projection of P′ in the orbital plane of PSP. $\chi $ is the scattering angle at P, while $\chi '$ the scattering angle at P′. $\gamma '$ is the angle at the Sun in the Sun-P′-PSP triangle. Panel b shows instead the projected points along a coronal streamer (magenta crosses) in the orbit plane of PSP. The points have a fixed $\gamma $, but a varying scattering angle $\chi $, as well as, varying elongation $\varepsilon $ along it.

Figure 2 shows the Thomson scattering geometry, used in our analysis in both 3D (panel a) and 2D (panel b) views. Specifically, panel a includes the Sun, PSP and a point P′ along a coronal ray in the |Sun-PSP| reference system. The coordinates for PSP and the feature P′ under investigation are defined relative to a reference frame $x$ and $y$, which lies in the same plane as PSP orbit. The origin of the reference frame is at the center of the Sun. With respect to that, the coordinates, radial distance, longitude and latitude, of the feature P′ are $r_{2}$, $\gamma $, and $\delta _{2}$ and for PSP are $r_{1}$, $\gamma _{\mathrm{PSP}}$, and $\delta _{1}$. For convenience we choose the direction of the reference axis, $x$, in the Sun-PSP direction, $\gamma _{\mathrm{PSP}} = 0$ and $\delta _{1} = 0$. P is defined as the projection of P′ in the orbital plane of the spacecraft, $\varepsilon $ the elongation of the projected feature, $\chi $ the scattering angle of the projected feature P. Panel b provides a top view of panel a and illustrates the scattering geometry for the case where the ray lies in the PSP orbital plane ($\beta = 0$). Additionally, panel b shows different tracked points along the coronal streamer (indicated by magenta crosses). In this scenario, it is important to note that these points share a fixed $\gamma $, while the scattering angle $\chi $ and elongation $\varepsilon $ vary along the streamer. The scattering angle $\chi $ reaches $90^{\circ}$ at the point P located at the TS (e.g., $\chi _{3} = 90^{\circ}$). When a ray is tangent to the TS ($\gamma = 90^{\circ}$), the scattering angle is close to $\chi = \pi -(\gamma +\varepsilon ) \approx 90^{\circ}$ for small elongations $\varepsilon $, i.e. when the feature is near the Sun. As $\varepsilon $ increases, $\chi $ decreases. On the other hand, for a ray at $\gamma = 60^{\circ}$, the scattering angle approaches $90^{\circ}$ only at larger elongations. However, since the feature is then located farther from the Sun, the electron density is reduced due to radial fall-off, and the corresponding brightness is lower compared to the case of $\chi \approx 90^{\circ}$ with $\gamma = 90^{\circ}$ near the Sun. This reasoning applies when the feature lies in the PSP orbit plane (i.e., when $\beta = 0$). If the feature is out of the orbit plane, the 2D geometry must be extended to a full 3D formulation of the scattering geometry, using the triangle defined by $\gamma '$, $\chi '$, and $\varepsilon '$. In this case, the variation of the scattering angle $\chi '$ follows the same formulation as a function of the elongation $\varepsilon '$ described above.

In fact, the angle $\chi '$ represents the scattering angle, i.e., the angle at the point P′ between the lines connecting it to the Sun and it to PSP, for a feature moving out of the plane of the spacecraft. It is defined as:

1 $$ \chi '=\pi -[\gamma '+\varepsilon '], $$

where $\gamma '$ is the angle at the Sun in the PSP-Sun-P′ triangle and $\varepsilon '$ the central angle.^2^ At the TS, the scattering angle, $\chi '$, for a point P′ is $90^{\circ}$. Considering a static approximation of the equations given in Nisticò et al. (2020), we compute the brightness of a coronal streamer along its central angle at discrete time steps using the following theoretical expression for each $i$-tracked point:

2 $$ B_{the,i}=\frac{C}{r_{1}^{2}\sin ^{2}\varepsilon '_{i}}[1-\cos ^{4}( \gamma '+\varepsilon '_{i})] $$

with $r_{1}$ $[\mathrm{m}]$ the distance of PSP from the Sun and the parameter $C$ defined as

3 $$ C=\pi B_{\odot }R^{2}_{\odot}\sigma _{T}nh. $$

$B_{\odot}$ is the Sun’s radiance ($B_{\odot}= 1~\mathrm{MSB} = 2.3\cdot 10^{7}~\mathrm{Wm}^{-2}\,\mathrm{sr}^{-1})$, $R_{\odot}$ is the solar radius of $R_{\odot}=6.96\cdot 10^{8}~\mathrm{m}$ and the total Thomson cross-section is defined by $\sigma _{T}=\frac{8\pi}{3}r^{2}_{e} = \frac{8\pi}{3}\big( \frac{e^{2}}{4\pi \varepsilon _{0}m_{e}c^{2}}\big)^{2}=6.65\cdot 10^{-29}~\mathrm{m}^{2}$, where $r_{e}$ is the classical electron radius. Finally, $n$ [$\mathrm{m}^{-3}$] is the plasma density number, and $h$ [m] the average column depth of the feature along the LOS. By measuring the brightness profile of the streamer ray over $\varepsilon '$ (see Section 2.1), and fitting that to the theoretical profile, the angle $\gamma '$ and the parameter $C$ can be determined.

Specifically, $\gamma '$ is varied in steps of 1° between 0° and 180° from PSP, and the parameter $C $ for several orders of magnitude between $0.1\cdot 10^{6}~\mathrm{MSB}\,\mathrm{m}^{2}$ and $1.0\cdot 10^{10}~\mathrm{MSB}\,\mathrm{m}^{2}$. We obtain the best agreement between the theoretical and observed brightness by selecting the combination of fitting parameters, namely C and $\gamma '$, that minimizes the residual:

4 $$ \sigma _{B} = \frac{1}{N} \sum _{i=1}^{N} \left | B_{\mathrm{the}, i} - B_{ \mathrm{obs}, i} \right |. $$

**Figure 3:**
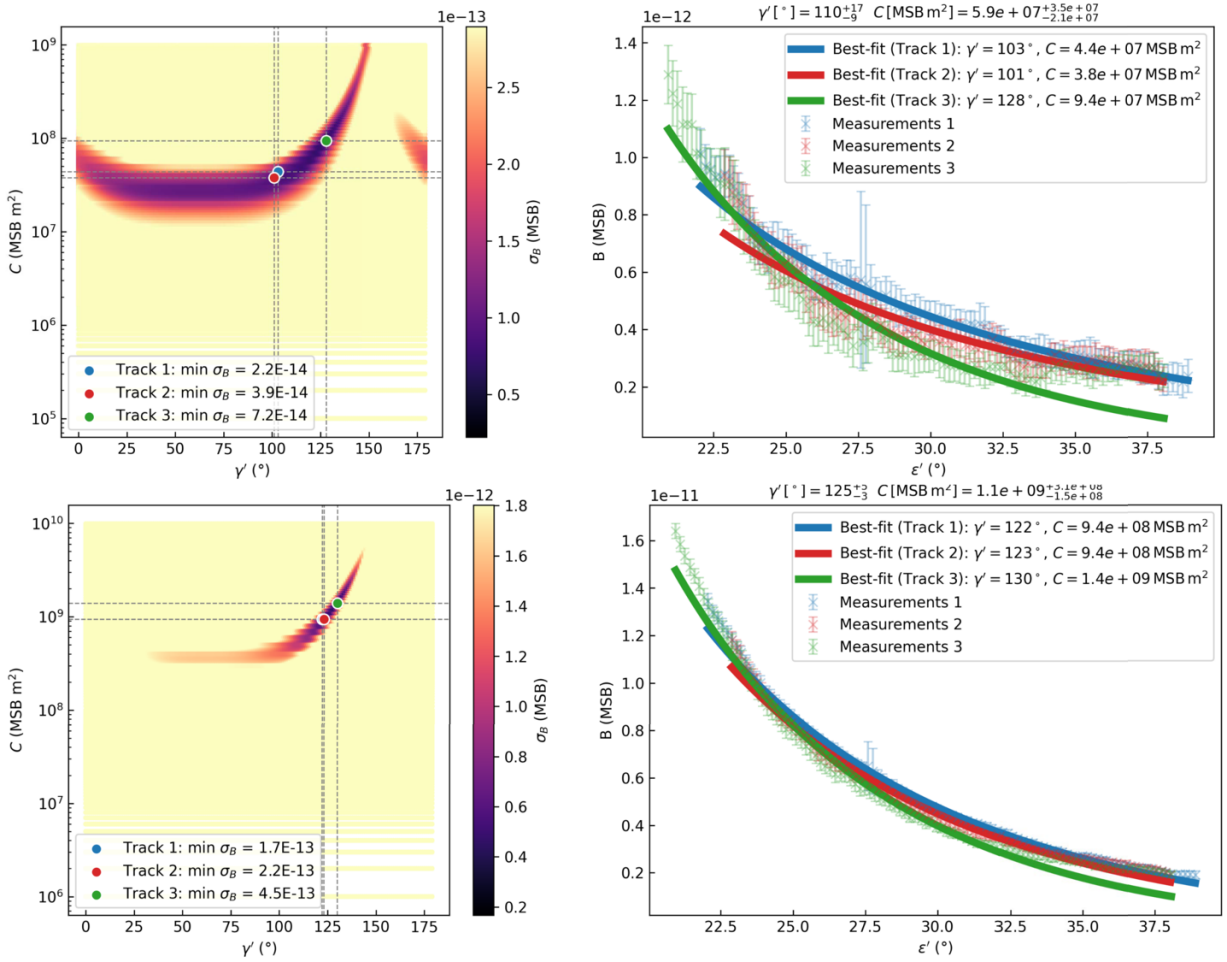
Once a ray is selected, it is tracked three times in the L3 data, for error calculation purposes, before the fitting is performed. The results for L3 (top row) and LX (bottom row) data are shown. Each row presents the same analysis steps for a different dataset. The left column is a color map showing the minimal residual of the three fits over the large set of parameters used. The best $C $ and $\gamma $ values according to the derived minimum value of $\sigma _{B}$ are shown respectively to the track as blue, re, and green circles. The right column shows a plot of the theoretical brightness profile over central angle calculated by using the $C$ and $\gamma $ values matching best the measured one for each track. The blue (red/green) crosses are related to the measurement of the first (second/third) one and the solid blue (red/green) line to the fitting. The error bars at each point are calculated as the standard deviation within a $7 \times 7$ pixel box centered on that point.

The residual of the fitting procedure is visualized in the left column of Figure 3 for the L3 dataset (first row) and LX dataset (second row), respectively. To estimate an error range for $\gamma '$ and $C$ we repeat the tracking and the fitting operations three times per each ray, using different start and end points. Hence, three different brightness profiles are derived for the same streamer that are then used to fit the theoretical profile. For each measured brightness profile, the fitting procedure determines the optimal values of $C $ and $\gamma '$ that minimize $\sigma _{B}$ (see the blue, red, and green dots overplotted on the colormap in the left column in Figure 3, corresponding to the best solution for tracks 1, 2, and 3 respectively). For simplicity, the $C $ value is assumed to be constant with distance from the Sun during the fitting procedure and is later interpreted as an average value that encapsulates information about the width of the streamer. Once the best parameters are found for each track, the measured and theoretical brightness profiles over central angle $\varepsilon '$ are compared for each track (see right column in Figure 3). The mean of the fitted values across the three tracks is taken as the best estimate, and the uncertainties are given by the maximum and minimum values among them, representing the range of possible solutions. Specifically, for the LX dataset the errors are one order of magnitude smaller since the brightness profiles are smoother (see Figure 1).

Once $\gamma '$ has been determined, the longitude $\gamma $ and latitude $\delta _{2}$ is obtained. The full mathematical derivation can be found in the Appendix. With that the BriLo technique allows to derive the positional, as well as physical, information of the streamer by using single-spacecraft measurements at one instance of time. The code developed to perform the tracking of the rays, the fitting of the measured brightness along them, and the error calculations is public and available on Github.^3^

### Validation of BriLo Using Triangulation

To assess the performance of the BriLo method we validate the derived results using triangulation as a completely independent method. For the triangulation at least two different vantage points need to be combined. We use coronagraph data from the Solar Terrestrial Relation Observatory (STEREO-A; Kaiser et al. 2008), namely COR2 (Howard et al. 2008), together with the Large Angle and Spectrometric Coronagraph (LASCO) C2 and C3 (Brueckner et al. 1995) on board the Solar and Heliospheric Observatory (SOHO; Domingo, Fleck, and Poland 1995). For each case study, we select at least two viewpoints that are well separated from each other. By performing the multi-spacecraft triangulation we obtain for each streamer the latitude and longitude.

**Figure 4:**
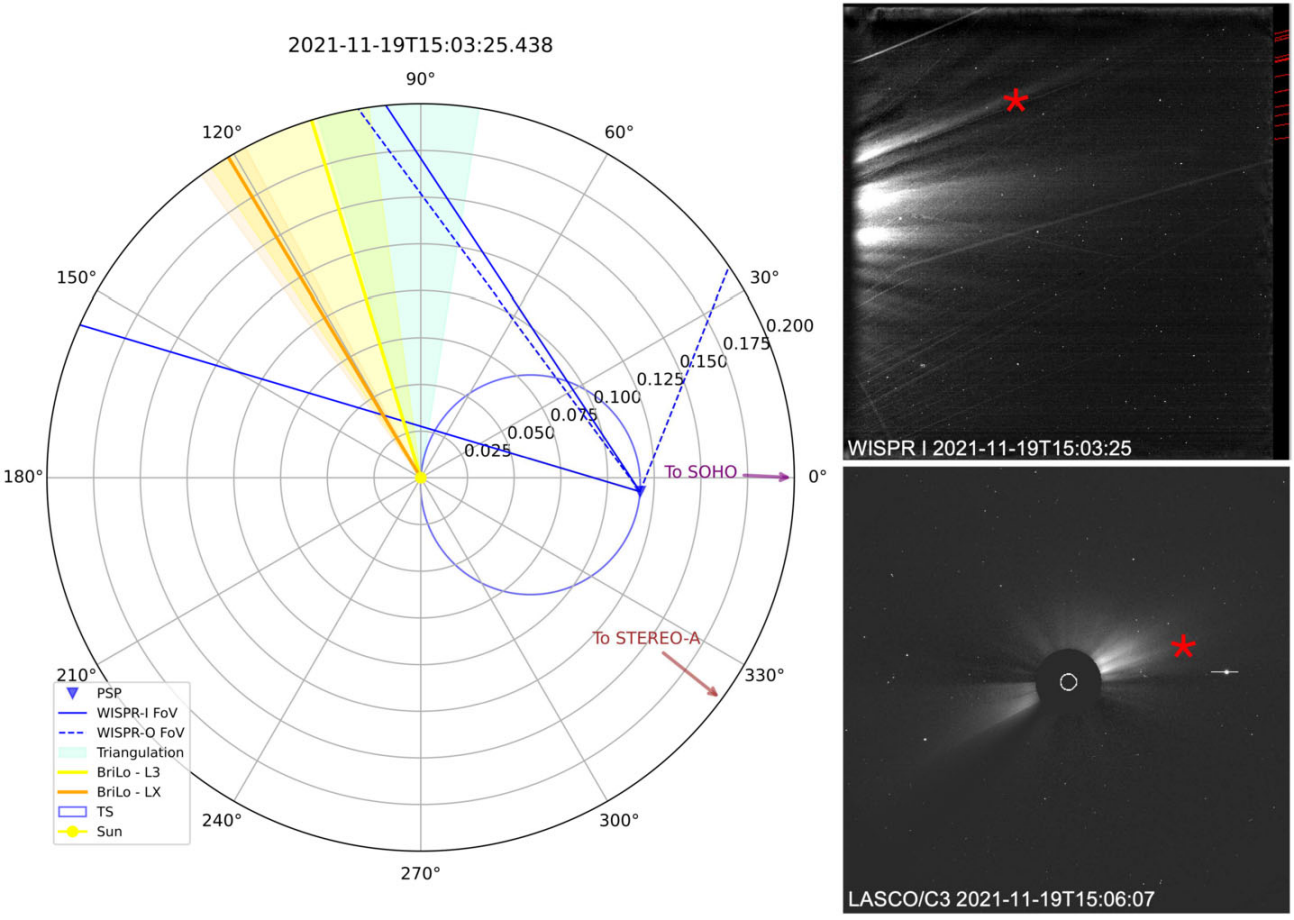
Derived longitude (latitude is not shown) for a helmet streamer using BriLo and the triangulation method for the event on November 19, 2021 (streamer number 5). The same coronal streamer is observed from two different viewpoints, PSP/WISPR-I (top right) and SOHO/C3 (bottom right). The red cross indicates the same feature. On the left, the spacecraft constellation in HGC for this event is shown. The FoV of WISPR-I (13°–53°) is indicated by the blue solid lines. The arrows show the direction to STEREO-A and SOHO. The inferred location of a coronal streamer is obtained by three methods: yellow (BriLo–L3), orange (BriLo–LX), and aquamarine (triangulation). Shaded areas represent angular uncertainties. The blue circle marks the TS for PSP/WISPR. Spacecraft directions (PSP, SOHO, STEREO-A) are indicated for reference.

For this purpose, we apply the scc_measure.pro routine available in the SolarSoft package in IDL. First, a point along the streamer is selected in one image (e.g., WISPR-I). The corresponding epipolar line, representing the possible 3D location of the point, then appears in the other viewpoint (e.g., SOHO/C3). By selecting the streamer where it intersects the epipolar line in these images, scc_measure.pro calculates the 3D position (see, e.g., Inhester 2006). Figure 4 shows an example of the results of the triangulation for streamer no. 5, together with a zoomed view of the spacecraft constellation for the event including a comparison on the solution of triangulation, in aquamarine, and BriLo method in yellow for L3 data and in orange for LX data. The shaded areas represent the uncertainties associated with the different methods and illustrate that even triangulation techniques can produce significant errors.

In summary, the uncertainty in the BriLo method is quantified by averaging the solutions obtained from each of the three separate tracks. For each track, the optimal solution corresponds to the one with the minimum residual error, as described in Section 2.2. The uncertainty in the triangulation results is determined by performing the triangulation using different combinations of spacecraft and at multiple points along the streamer, followed by averaging the outcomes. Different spacecraft combinations may produce slightly varying triangulation results, which are reported separately. In general, a separation angle greater than $20^{\circ}$ between the planes of sky of the two viewpoints is recommended to ensure reliable results.

Although the longitudinal separation between PSP and SOHO is relatively small for streamer no. 5, the FoV of WISPR is significantly offset from that of SOHO’s coronagraph. This offset results in a favorable separation angle between their respective planes of sky, enabling reliable triangulation. On the other hand, the spacecraft constellation for streamer no. 7 (Figure 14 in the Appendix) shows a very small separation between the planes of sky of the different instruments, resulting in less reliable triangulation. A key advantage of BriLo is that it relies solely on data from a single spacecraft, avoiding this geometric limitation. The spacecraft constellation and the triangulation from multiple viewpoints are provided in the Appendix for each study example.

## Results

The BriLo method was applied to ten coronal streamers observed by WISPR-I during various PSP encounters (E1, E7, E8, E9, E10, E11, E15, and E17) between 2018 and 2023. Rays were manually selected through visual inspection of WISPR-I data, aiming to identify a set of bright, collimated and well-defined ray-like structures. Table 1 lists, for each studied ray, the observation dates, PSP locations, and the results from the BriLo method, as well as the corresponding triangulation results. In fact, all streamers were observed from at least two spacecraft with different vantage points, which allowed to perform triangulation to validate our results (see Figures 8–17 in the Appendix).

A key advantage of BriLo is that it relies exclusively on data from a single spacecraft, thus avoiding the geometric limitations imposed by the need for sufficient spacecraft separation to perform reliable triangulation. For the time range studied, PSP was at varying heliocentric distances between 13 and 36 $\mathrm{R}_{\odot}$. As a consequence, the FoV of WISPR-I covered different physical distances^4^ (see column 8 in Table 1).

Table 1 shows that the analyzed coronal streamers have $\gamma '$ equal to or greater than 90° for the majority of events, with a specific range of 85° to 129°. Based on the spacecraft location and the TS given for each case study (see panel d in Figures 8–17 in the Appendix), we notice that the studied streamers are located near or beyond the tangential point where the TS connects to the Sun. This indicates that these streamers lie on or beyond the TS of PSP, i.e., near the plane-of-sky (see Section 2.2).

**Figure 5:**
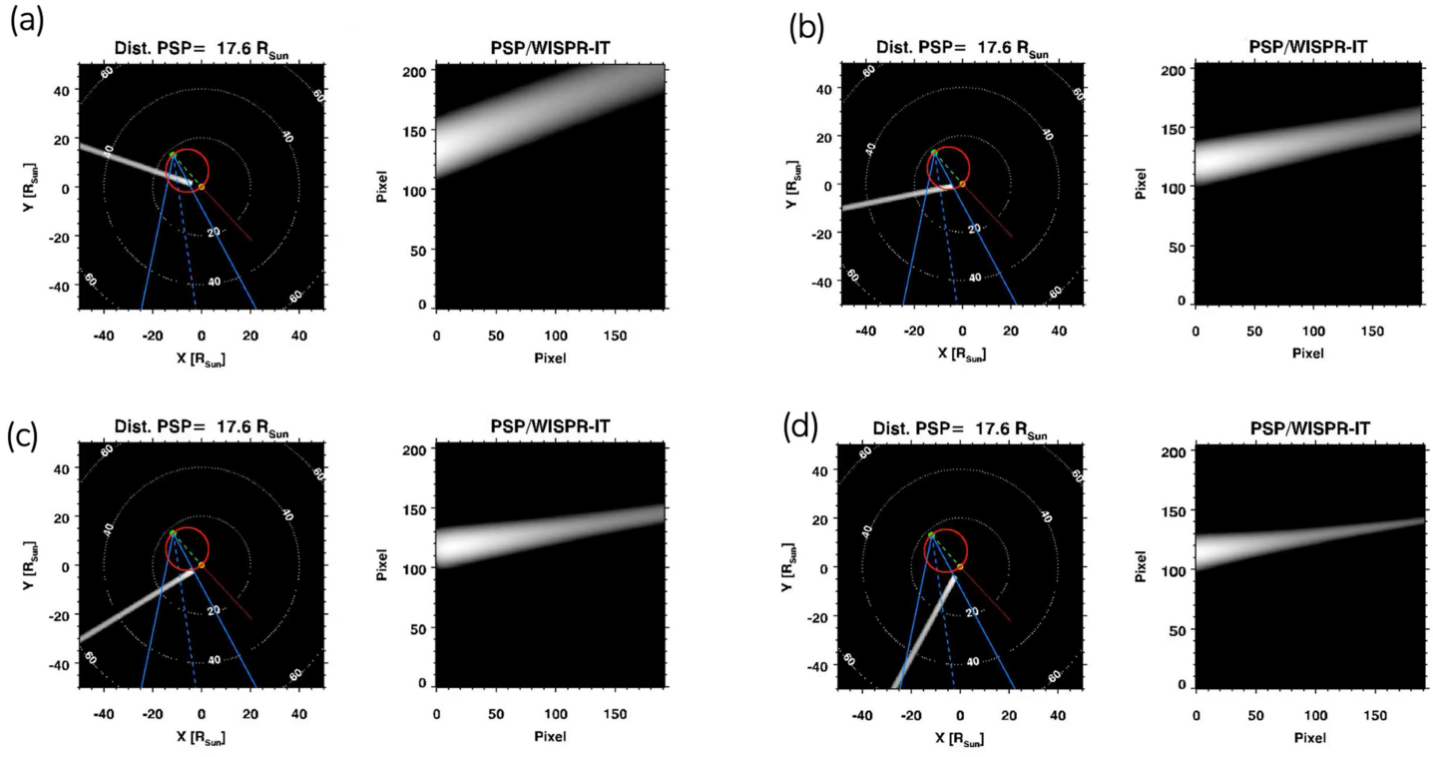
Raytracing simulations for a coronal streamer located at different longitudes on the Sun, hence, having a different angle as viewed from PSP in the WISPR-I field-of-view. The latitude $\delta _{2}$ is fixed at 10°. (a) 30° from PSP in longitude $\gamma $. (b) 60° from PSP in longitude $\gamma $. (c) 90° from PSP in longitude $\gamma $. (d) 120° from PSP in longitude $\gamma $.

This may be related to our selection of streamers for analysis, specifically, distinct, narrow, sharply delineated, ray-shaped structures. As for the brightness criterion, this naturally led us to selecting streamers close to the TS. As for the well-defined ray-like structure criterion, this can be explained by performing simple ray-tracing simulations using a model originally presented in Thernisien, Howard, and Vourlidas (2006) and later adapted by Nisticò et al. (2020) for PSP orbit.^5^ We simulated rays having the same density profile^6^ over distance and spanning a wide range of longitude. The results are shown in Figure 5 for a longitudinal range of $\gamma $ ranging between 30°–120° and a fixed latitude of $\delta _{2} = 10^{\circ}$. It is evident that rays observed from larger longitudinal separations relative to PSP appear narrower and more pointy, whereas those observed from positions closer in longitude tend to be broader and less defined. Closer-in views cause the rays to appear broader as a result of the LOS integration of the optically thin emission. Hence, our choice of streamers has a selection bias towards well-defined ray-like features, lying further away from the PSP, opposite to broad and blurry ones, lying closer to the PSP.

The BriLo method was applied to both L3 and LX data products. L3 and LX data products show good agreement in deriving streamer directionality with differences of 2 – 30° in longitude and 1 – 6° in latitude. Both datasets yield longitude and latitude estimates broadly consistent with triangulation results. Nevertheless, we notice that the BriLo solutions strongly deviate from those of triangulation for rays 7, 8, and 9. As explained in Section 2.3, the triangulation of ray 7 is not reliable because of the small separation in between the plane of the sky of the instrument used. For ray 8 and 9, we instead notice that BriLo gives comparable results to triangulation using L3 data but gives large discrepancies when using LX data. The two rays are in WISPR FoV at the same timestamp (Table 1). A closer look at the event shows some brightness variability along the rays near the studied time, likely due to a preceding CME in the South (cf. Figures 15 and 16). On the one hand, BriLo performance is expected to decrease when streamers exhibit brightness variations or deviate from their radial extension, presumably due to shock interactions. Therefore, it is recommended to focus on time periods during which streamer features remain stable. On the other hand, it is interesting to note that results for BriLo applied to LX data are more strongly affected by streamer brightness variability than L3.

For the calculated $C$ values we find a range between $1.7\times 10^{7}$ and $1.7\times 10^{8}$ MSB m^−2^ when BriLo is applied to the L3 data, while derived $C$ values range between $1.8\times 10^{8}$ and $2.2\times 10^{9}$ MSB m^−2^ when BriLo is applied to the LX data. The differences in the $C$ value clearly reveal the effects of WISPR image reduction techniques for the K- and F-coronal components (see more details in Section 2). Hence, this leads to differences between the longitude, latitude, and $C$ values obtained with the two different datasets.

**Figure 6:**
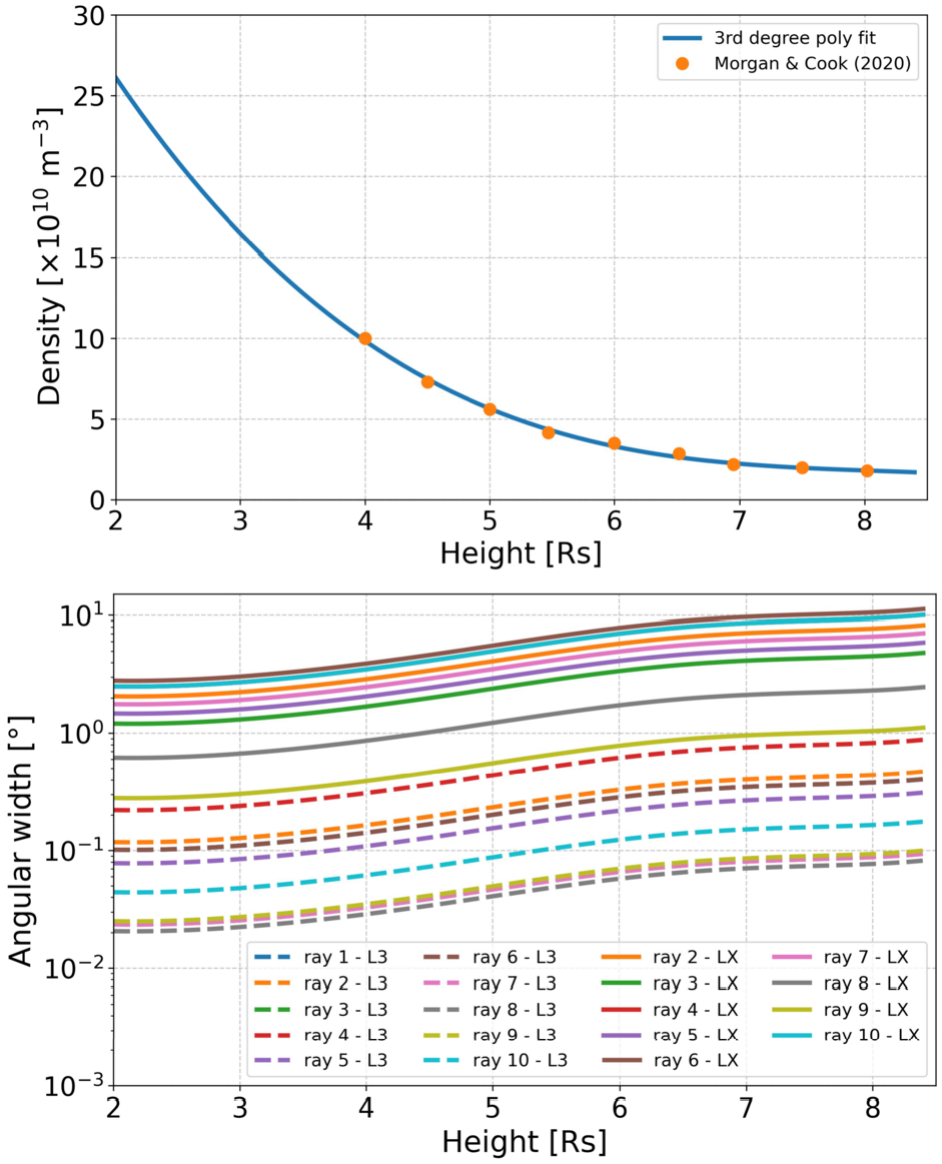
Using the density values from Morgan and Cook (2020, see their Figure 8) we apply a polynomial fit to get a density profile, density versus height. Under the assumption of $C\sim$ const, and its interpretation as an average value, we apply Equation 3 in order to estimate for a streamer its column width, $h$. We also assume that the majority of the brightness signal comes from the streamer. From this we derive the angular width of the streamer (see text for more details).

The values of $C$, assuming it to be an average value over the streamer structure, have also implications on the structure width. Differences by one order of magnitude, as found in this study, lead to differences when calculating the streamer width $h$ (see Equation 3). Applying Equation 3 with $C\sim n \cdot h$, we can use plasma density profiles available in the literature for streamers to relate it back to their column width. Assuming a sheet-like streamer geometry that expands in the longitudinal direction ($\gamma '$) with increasing heliocentric distance, while maintaining a narrow, stalk-like appearance when viewed edge-on, we compute the LOS-integrated column width. This width is approximated by the chord length—i.e., the line segment connecting two points on a circular arc with a radius $r_{2}$ (in units of $\mathrm{R}_{\odot}$)—which is then converted to an angular width. Figure 6 shows in the top panel an average streamer density profile over height based on the density values given in Morgan and Cook (2020) using a tomographic approach. Calculating $h$ with the simple method above using the given density profiles and derived $C$ values from this study, from LX data we find streamer widths in the range of several up to ten degrees over a distance range from 4 – 8 $\mathrm{R}_{\odot}$. Similar results are also reported by Morgan and Cook (2020). From L3 data we derive streamer widths in the sub-degree range. From this, we may conclude that the results for the $C$ parameter in BriLo using L3 data might be underestimated compared to those obtained with LX data. This gives important hints on the image processing techniques applied to WISPR data.

We then related the derived streamer locations to their solar source regions, by overploting BriLo-L3 and -LX results together with triangulation results on a synoptic magnetogram. For this we use data from the Helioseismic and Magnetic Imager (HMI; Scherrer et al. 2012) on board the Solar Dynamics Observatory (SDO; Pesnell, Thompson, and Chamberlin 2012). The synoptic map also includes the extrapolated HCS, calculated from the PFSS model (Altschuler and Newkirk 1969), using the finite difference method. This approach numerically solves the Laplace equation for the scalar potential by using a staggered grid configuration for the magnetic field. This setup guarantees that the magnetic field remains divergence-free and curl-free to within floating-point precision in the discrete model. Additionally, the model accurately reproduces the input magnetogram. To estimate the structure of the HCS, we computed it using a source surface height at 2.5 $\mathrm{R}_{ \odot}$—defined as the radial boundary where the magnetic field is constrained to be radial.

**Figure 7:**
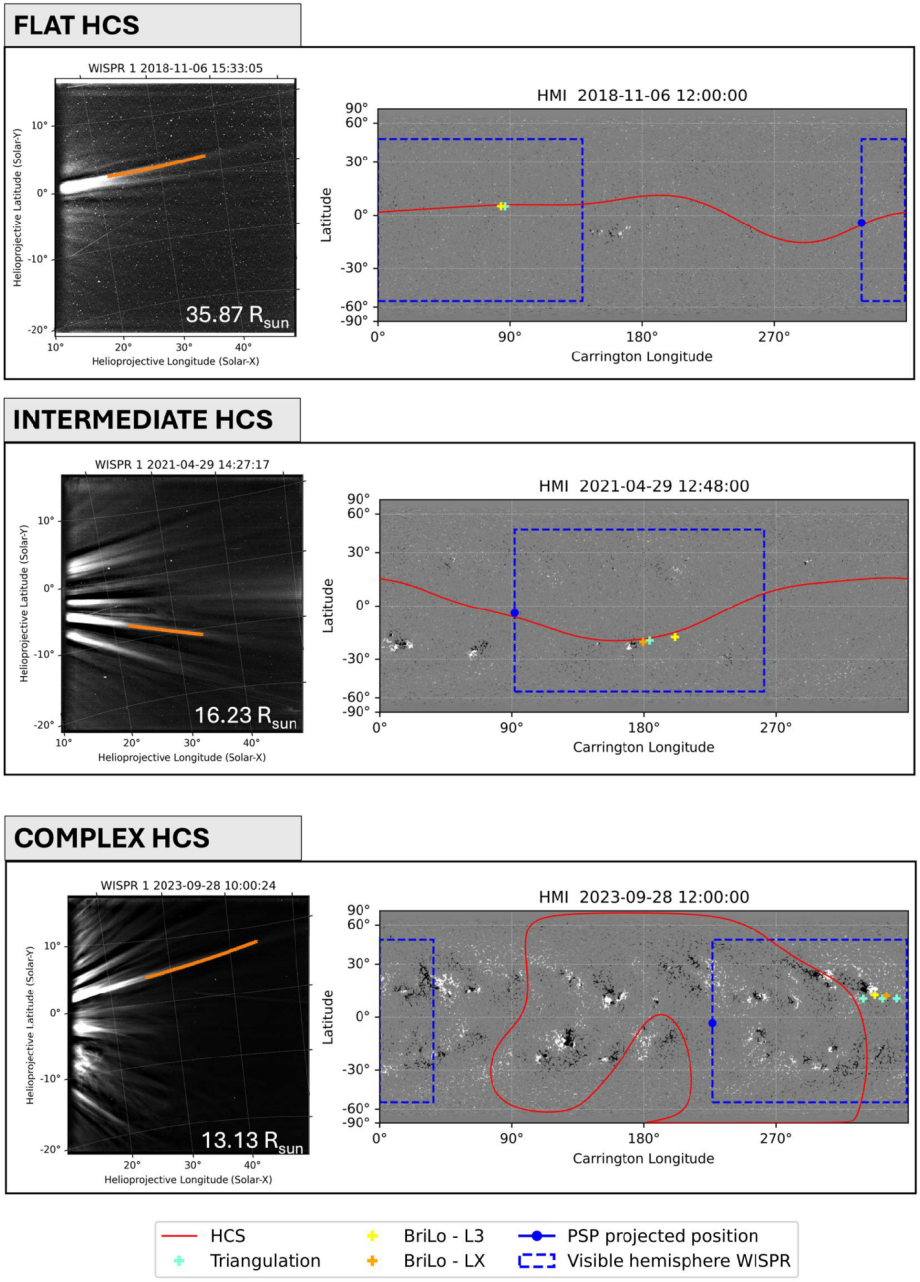
Examples of collection of streamers observed in the FoV of WISPR-I in three different HCS orientation, as described for each studied ray in Table 2. Shown from top to bottom are examples of flat, intermediate, and complex HCS within the WISPR FoV. We applied BriLo to the tracked orange ray in WISPR-I and compared the results: L3 in the Carrington map (orange cross), LX (yellow cross), and triangulation (aquamarine crosses). Synoptic magnetograms from HMI, including HCS profiles with a neutral line at 2.5 $\mathrm{R}_{\odot}$, were used for context. The dashed blue box indicates the FoV of WISPR-I. A longitudinal range of 180° was considered to account for coronal rays entering the FoV at any location, along with a latitudinal extent of approximately $\pm 50^{\circ}$.

The shape of the HCS changes over the solar cycle, becoming flatter during solar minimum and more warped during solar maximum conditions (see, e.g., Riley et al. 2006). Figure 7 shows a selection of 3 different orientations of the HCS within the WISPR-I FoV identified among the studied events and classified as either flat (low north-south extension), intermediate (medium north-south extension), or complex (i.e., high north-south extension or including magnetic islands). For each orientation, an example is provided illustrating how coronal streamers appear in the WISPR-I FoV under different HCS scenarios. Depending on how strongly warped the HCS is, individual streamers populate narrower and wider latitudinal ranges. Hence, particularly during solar minimum (e.g., top panel of Figure 7), streamer brightness profiles can be affected by LOS integration due to overlapping streamers at the same latitude, whereas approaching the solar maximum, the rays are distributed in a fan-like pattern across latitude (see lower panel of Figure 7). In general, from the comparison to the HCS we obtain that all streamers are found to be either located along the HCS or close to active regions. In the Appendix, these maps are shown individually for each coronal ray studied.

**Figure 8:**
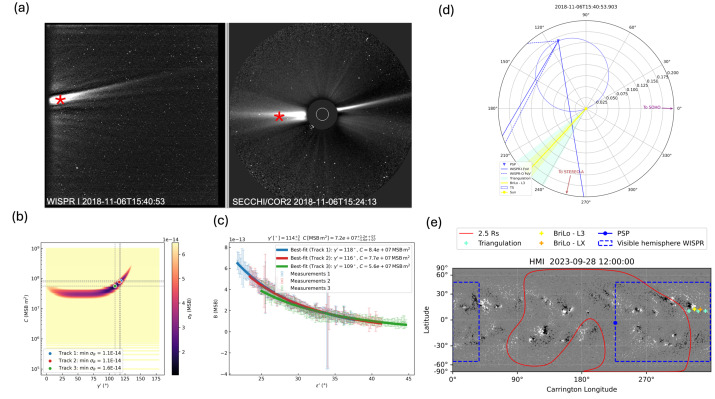
Summary plots for the results derived for coronal streamer no. 1. (a) Identification of the streamer through triangulation techniques using at least two viewpoints. (b) Scatter plot showing the result from the BriLo method, using L3 WISPR-I data, for various combinations of $\gamma $ and $C$. The color bar indicates the minimum residual for each set of tested parameters. The colored dots (blue, red, green) mark the best fitting parameter combination for each of the track (1, 2, 3) performed. (c) Brightness profile of the streamer and best-fit curve for each of the track. The average of the results of the three fits, and their errors, are shown in the top part of the figure. (d) Spacecraft constellation showing the FoV of WISPR in blue and the location of the streamer based on the results from BriLo using L3 data (yellow), using LX data (orange) when available, and using triangulation (aquamarine). Shaded areas represent angular uncertainties on the different methods. The arrow indicates the direction to STEREO-A and SOHO. (e) Synoptic HMI magnetogram including the location of the HCS considering a neutral point line at 2.5 $\mathrm{R}_{\odot}$ and the derived location of the coronal streamer no. 1. The results of BriLo using L3 WISPR-I data are shown by the yellow cross, the results of the LX WISPR-I data by a orange cross (only when this data product is available). The results using triangulation techniques from multiple combination of instruments and viewpoints are indicated with the aquamarine cross. The blue box in panel e outlines the region from which rays could potentially enter WISPR FoV, based solely on its geometric FoV.

**Figure 9:**
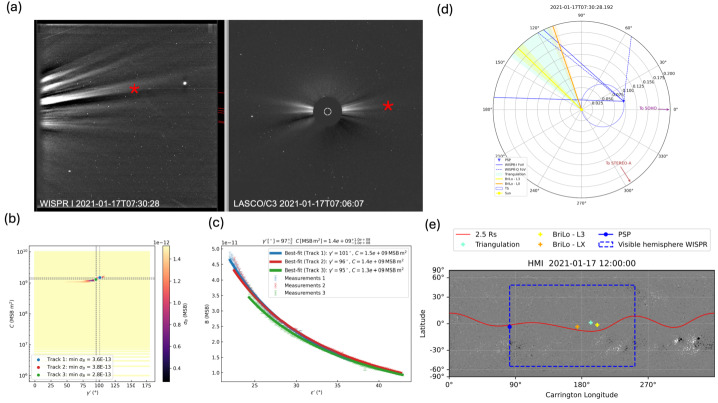
Same as Figure 8 but for coronal streamer no. 2.

**Figure 10:**
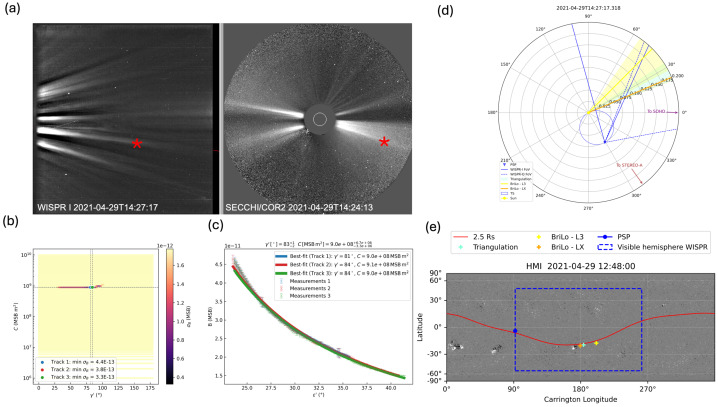
Same as Figure 8 but for coronal streamer no. 3.

**Figure 11:**
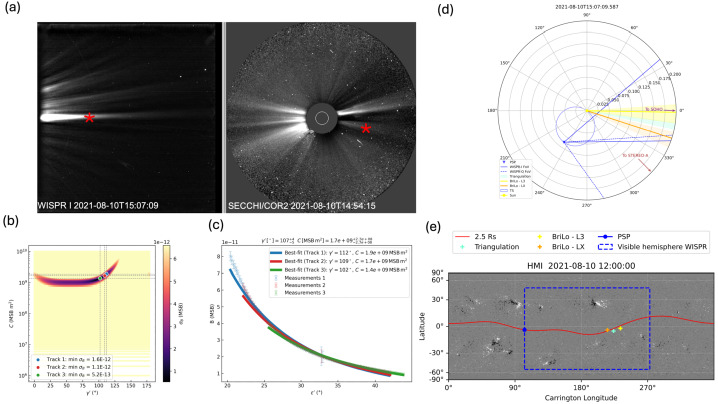
Same as Figure 8 but for coronal streamer no. 4.

**Figure 12:**
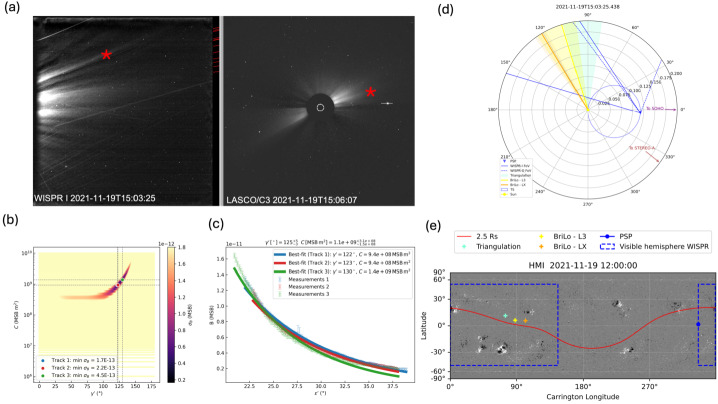
Same as Figure 8 but for coronal streamer no. 5.

**Figure 13:**
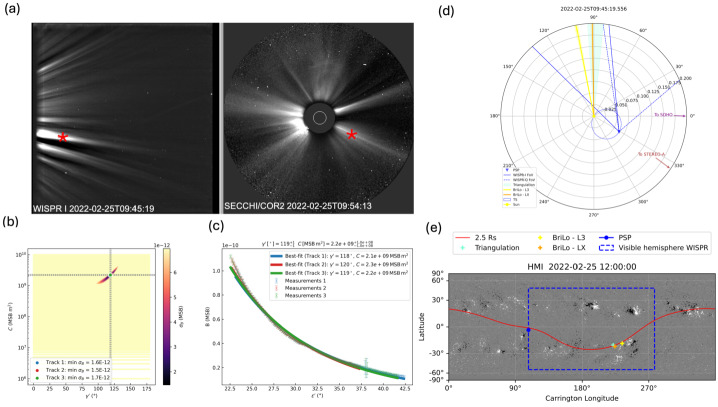
Figure 8 but for coronal streamer no. 6.

**Figure 14:**
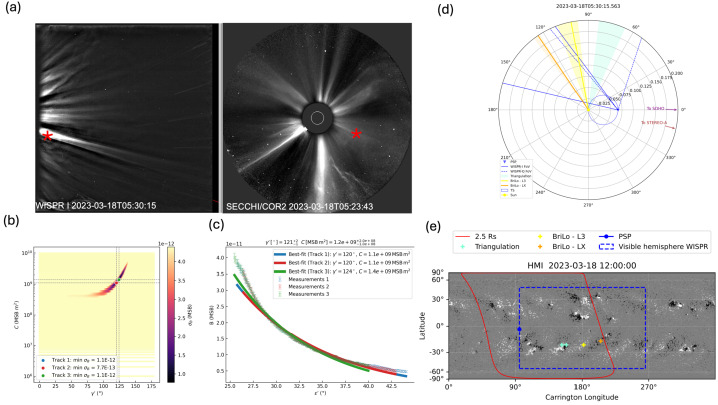
Same as Figure 8 but for coronal streamer no. 7.

**Figure 15:**
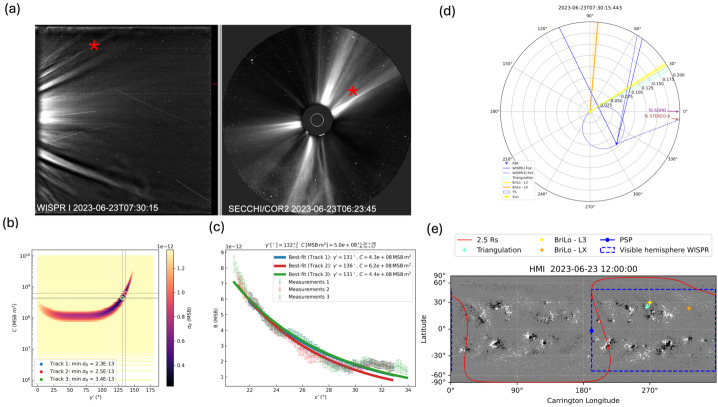
Same as Figure 8 but for coronal streamer no. 8.

**Figure 16:**
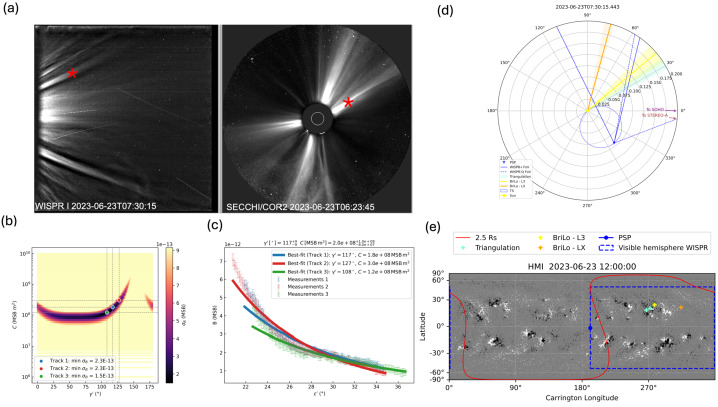
Same as Figure 8 but for coronal streamer no. 9.

**Figure 17:**
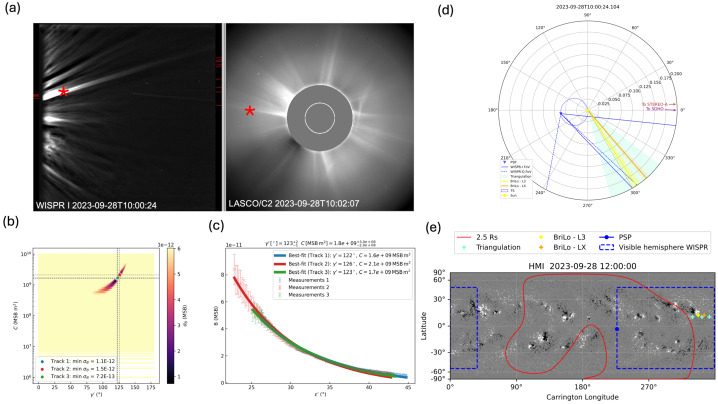
Same as Figure 8 but for coronal streamer no. 10.

To find out more about the solar source regions of the tracked coronal streamers we also visually classified them either into helmet streamers (HS) or pseudostreamers (PS). Following the findings from Wang et al. (2007), we identified three out of ten streamers in SOHO/LASCO coronagraph images as PS, based on the absence of a dome above $\sim 2~\mathrm{R}_{\odot}$. The plots in panel e for each streamer in Figures 8–17 show that most HS are located close to the HCS. Two out of the three PS are located close to ARs. Table 2 summarizes the orientation of the HCS for each example, the origin of the coronal ray (either HCS or active region, AR), and the visual classification as HS or PS.

**Table 2:** Overview of the HCS shape on the observation dates of rays no. 1 – 10 under analysis, including the association of the origin of each ray with either the HCS or active regions (AR) which is marked with ‘x’. The type of streamer is given as helmet streamer (HS) or pseudo-streamer (PS).

No.	Slope HCS	HCS	AR	type
1	flat	x	–	HS
2	intermediate	x	–	HS
3	intermediate	x	–	HS
4	intermediate	x	–	PS
5	intermediate	x	–	HS
6	intermediate	x	–	HS
7	complex	−	x	PS
8	complex	−	x	PS
9	complex	−	x	HS
10	complex	x	x	HS

## Discussion

BriLo is a newly developed technique designed to infer the 3D location (latitude and longitude) and physical properties of coronal streamers, encapsulated in the parameter $C$, which represents the electron density ($n_{e}$) integrated over the width ($h$). After tracking individual rays in F-corona–removed data products, we derive brightness profiles and apply Thomson scattering theory, as described in Section 2.1, to extract the relevant information through a fitting procedure. This method has been applied to ten randomly chosen, bright and collimated coronal rays well-observed by WISPR-I during encounters 1 through 17.

The BriLo derived results (see Table 1) were verified by using independently triangulation. A key advantage of BriLo is that it relies exclusively on data from a single spacecraft, thereby avoiding the geometric limitations associated with the requirement of sufficient spacecraft separation for reliable triangulation. Supported by raytracing simulations, we found that selection criteria applied for our study, namely bright, well-defined streamers, led us to choose those located at larger angular distances from the spacecraft, near or beyond the TS of PSP.

BriLo has been tested on both L3 and LX level WISPR-I data products for rays 2 – 10, each of which applies a procedure to remove the contribution from the F-corona. LX data products were not available for the first three encounters of PSP, hence no data is available for ray 1. We notice that the results obtained from the two datasets differ by about 2 – 30° in longitude, 1 – 6° in latitude, and by one order of magnitude in the derived $C$ values. As illustrated in Figure 1, this discrepancy arises because the brightness profiles along the same ray differ by at least an order of magnitude between the LX and L3 images.

This variation highlights a key challenge: the observed signal—whether K- or F-corona—results from integration along the LOS. Consequently, any procedure designed to remove the F-corona is affected by both the method used to estimate the background and the coronal environment along the LOS, leading to variations in the results obtained from the LX or L3 datasets. For example, if the streamer is located in front of or behind a coronal hole extension, superposition effects are reduced, and LX and L3 results may tend to be more similar.

In general, we obtain that the longitude and latitude estimates obtained using the L3 and LX BriLo technique are broadly consistent with those from triangulation for all the studied rays, with discrepancies of maximum 30° in longitude and 4° in latitude.

Once the location of the streamers has been determined, we compared them to the global magnetic field information using synoptic magnetograms from the HMI on board the Solar Dynamics Observatory (SDO). Besides giving information on the source region, we also checked the orientation and closeness of the streamer to the HCS. We identified various slopes of the HCS, summarized in Table 2, and categorized them as flat, intermediate, and complex (see also Figure 7). In general, we obtain that most of the rays visually classified as HS are located in the proximity of the HCS, while the PS are mostly near by ARs. We note that for a flat HCS multiple structures related to the HCS may overlap, which may influence the extracted brightness profiles due to LOS integration effects.

Finally, using the average streamer density profile from Morgan and Cook (2020) and applying the $C$ values from this study, we find streamer angular widths in the sub-degree range for L3 and of several degrees for LX data products. In that respect, brightness profiles from LX data are more feasible compared to L3. This highlights the discrepancy in the image reduction techniques.

## Conclusion and Outlook

We summarize our main findings:

- Using the Thomson scattering theory, BriLo enables to derive from single spacecraft WL imagery the longitudinal and latitudinal position of streamer structures, eliminating the need for wide spacecraft separation required by triangulation methods.
- BriLo has been applied to different WISPR data products (L3 and LX), resulting in rather different performance:
  - We observe that results from the two datasets differ by 2 – 30° in longitude, 1 – 6° in latitude, and by roughly one order of magnitude in the derived $C$ values.
  - For most cases, L3 and LX data products show good agreement in deriving streamer directionality relative to triangulation, with deviations up to 30° in longitude and 4° in latitude.
  - Due to the order-of-magnitude difference in absolute brightness between L3 and LX, $C$ values from LX data yield more realistic streamer widths (several up to ten degrees, consistent with Morgan and Cook 2020), whereas L3 data produce unrealistically narrow widths in the sub-degree range.
  - It is not possible to perfectly separate the F-corona from the static K-corona along the entire LOS in WL images. F-corona removal techniques can be used to estimate and correct for the F-corona, but their results depend on the background estimation and the coronal environment, which can lead to differences between LX and L3 datasets.
- Most of streamers identified as helmet streamers were found in the vicinity of the HCS while pseudostreamers around active regions.

As a next step, we aim to apply BriLo to data from 1 AU (e.g., COR2, C2/C3, Aditya, the Polarimeter to UNify the Corona and Heliosphere (PUNCH)) in order to derive brightness profiles and investigate both LOS integration and instrumental effects. Future developments of BriLo will include a parametrization of $C$, allowing it to vary with radial distance. This will enable the estimation of radially dependent streamer widths, rather than a single average value, thereby permitting more detailed interpretations of their variations. These refinements, combined with coronal density maps from, e.g., Metis on board Solar Orbiter (Antonucci et al. 2020), might turn BriLo into a powerful diagnostic tool for coronal streamers. In addition, BriLo shows strong potential as a tool for testing and validating WL imaging.

## Data Availability

In this study, we used publicly available data from the Wide-Field Imager for Parker Solar Probe (WISPR/PSP), the Solar and Heliospheric Observatory (SOHO), and the Solar Terrestrial Relations Observatory Ahead (STEREO-A). WISPR data are available at: wispr.nrl.navy.mil/wisprdata/ SOHO data are available at: https://lasco-www.nrl.navy.mil/index.php?p=get_data STEREO-A data are available at: https://secchi.nrl.navy.mil/get_data The BriLo python pipeline is publicly available at https://github.com/gretacappello/BriLo.

## Appendix A: Geometrical Derivation

Considering the 3D geometry schematic in Figure 2 panel a, we first apply the sine theorem to the Sun-P$_{i}'$-PSP triangle for each point $i$ along the streamer:

5 $$ \frac{r_{2,i}}{\sin\varepsilon '_{i}}=\frac{r_{1}}{\sin\chi _{i}}= \frac{|P'-\mathit{PSP}|_{i}}{\sin\gamma '_{i}}, $$

obtaining that $|P'-\mathit{PSP}|_{i} = \frac{r_{2,i} \sin\gamma '_{i}}{\sin\varepsilon '_{i}}$, where $r_{2,i} = r_{1} \frac{\sin\varepsilon '_{i}}{sin\chi '_{i}}$. Applying then the Pythagorean theorem to the Sun-P_*i*_-P$_{i}'$ and PSP-P_*i*_-P$_{i}'$ triangle, we derive that $|P-P'|_{i} = |P'-\mathit{PSP}|_{i}\sin\beta _{i} = r_{2,i} \sin\delta _{2,i}$, then we can therefore express the latitude of the ray as:

6 $$ \sin \delta _{2,i} = \frac{\sin \gamma ' \sin \beta _{i}}{\sin \varepsilon '_{i}} = \frac{\sin \gamma ' \sin \beta _{i}}{\sin (\arccos (\cos \beta _{i} \cos \varepsilon _{i}))}, $$

which admits two possible solutions:

7 $$ \delta _{2,i,a} = \arcsin \Bigg( \frac{\sin \gamma ' \sin \beta _{i}}{\sin (\arccos (\cos \beta _{i} \cos \varepsilon _{i}))}\Bigg), \delta _{2,i,b}' = \pi - \delta _{2,i,a}. $$

Between these two solutions, one is always greater than $90^{\circ}$. We discard the solution larger than $90^{\circ}$, as this angle corresponds to the latitude of the rays. Applying the sine theorem to the Sun-PSP-P_*i*_ triangle, we obtain:

8 $$ \frac{|\mathit{Sun}-P|_{i}}{\sin\varepsilon _{i}}= \frac{|P-\mathit{PSP}|_{i}}{\sin\gamma _{i}}. $$

Hence, we derive that:

9 $$ \sin\gamma _{i} = \frac{|P-\mathit{PSP}|_{i} \sin\varepsilon _{i}}{|\mathit{Sun}-P|_{i}} , $$

where $|\mathit{Sun}-P|_{i}=r_{2,i} \cos\delta _{2,i}$ and $|P-\mathit{PSP}|_{i}=|P'-\mathit{PSP}|_{i}\cos\beta _{i}$. The two solutions are then:

10 $$ \gamma _{i,a} = \arcsin\Big( \frac{\sin \gamma '_{i} \cos \beta _{i} \cos \varepsilon _{i}}{\sin \varepsilon '_{i} \cos \delta _{2,i}} \Big),\quad \gamma _{i,b} =\pi -\gamma _{i,a}. $$

Of the two solutions, we choose the $\gamma _{i}$ that is closest to the $\gamma '$ derived from the fitting procedure.

We treat $\gamma $ and $\delta _{2}$ as vectors along the ray. Although these angles should remain constant along the ray, small numerical variations can occur when computing them at different points. To ensure consistency, we adopt the first element of each array as the representative value. To convert $\gamma $ and $\delta _{2}$ into the HGS frame, we first define the unit vector $\mathbf{u}$ along the ray in the Sun–PSP reference frame:

11 $$ \mathbf{u} = \cos \delta _{2} \cos \gamma \,\mathbf{x} + \cos \delta _{2} \sin \gamma \,\mathbf{y} + \sin \delta _{2} \,\mathbf{z}, $$

where

$$ \mathbf{x} = \frac{\mathbf{r}_{\mathrm{1}}}{\|\mathbf{r}_{\mathrm{1}}\|}, \quad \mathbf{z} = \frac{\mathbf{r}_{\mathrm{1}} \times \mathbf{v}_{\mathrm{1}}}{\|\mathbf{r}_{\mathrm{1}} \times \mathbf{v}_{\mathrm{1}}\|}, \quad \mathbf{y} = \mathbf{z} \times \mathbf{x}. $$

Once the PSP-centered orthonormal triad $(\mathbf{x}, \mathbf{y}, \mathbf{z})$ is defined from $\mathbf{r}_{\mathrm{1}}$ and $\mathbf{v}_{\mathrm{1}}$ in Heliocentric Inertial (HCI) coordinates, it is then transformed into the HGS frame using the Python conversion routines.^7^

## Appendix B: Additional Figures

We show in Figures 8 to 17 summary plots for the derived results from triangulation, BriLo, relation of streamer location to the spacecraft position and relation of streamer location to the magnetic field and HCS. In Figure 8 panels b-c we report the solution of BriLo using the L3 dataset (since no LX data product is available for early PSP encounters), for streamers 2 – 10 (see Figures 9–17 panels b-c) we instead report the solution of the LX dataset.

## Footnotes

1 A PSP solar encounter is defined as the segment of the PSP orbit when the spacecraft is within 0.25 AU from the Sun.

2 The central angle ($\varepsilon '$), also referred to as the solar elongation, is computed using the spherical law of cosines. Thus, for this article, with the angles defined in Figure 2, $\varepsilon ' = \arccos \!\left (\sin (\mathit{lat}_{1})\sin (\beta ) + \cos (\mathit{lat}_{1}) \cos (\beta )\cos (\Delta \mathit{lon})\right )$, where $\mathit{lat}_{1}$ and $\mathit{lon}_{1}$ are the Sun’s reference latitude and longitude (typically 0,0 for the solar center), $\beta $ and $\varepsilon $ are the helioprojective latitude and longitude of the pixel, and $\Delta \mathit{lon} = \varepsilon - \mathit{lon}_{1}$. In the HPC system, solar elongation is often equated with the longitude coordinate, but this holds only when the latitude is zero; when the latitude is involved, the central angle formula accounts for the pixel elevation above or below the equatorial plane.

3 https://github.com/gretacappello/BriLo.

4 The range of distances spanned by the tracked points along the ray in each case study is calculated from the distance of PSP to the Sun and the central angle of each point, using the relation $r_{2} = r_{1} \frac{\sin \varepsilon '}{\sin \chi '}$.

5 The software package together with a user guide is available under https://zenodo.org/records/3478624.

6 For this simulation, we use a ray following a cylindrical coronal density model (model 17 of the ray-tracing tool), which employs the coronal density profile of Hayes, Vourlidas, and Howard (2001), expressed as $N_{e}(r) = \sum _{k} \alpha _{k} r^{-k}$, where we use the default coefficients $\alpha _{k}$ as $\alpha _{1} = 6.06 \times 10^{5}$, $\alpha _{2} = -1.59 \times 10^{7}$, $\alpha _{3} = 1.66 \times 10^{8}$, $\alpha _{4} = -4.7 \times 10^{8}$. This model includes an exponential decay term $\exp (- (d / d_{0})^{2})$, where $d$ is the perpendicular distance from the symmetry axis and $d_{0} = 0.4~\mathrm{R}_{\odot}$ represents the streamer half-thickness. All rays are simulated using these default coefficients, while the Carrington longitude and latitude are varied to explore different LOS geometries.
